# Research on the Evolution Law Physical Short Fatigue Crack and Tip Deformation Fields during Crack Closure Process of the Q&P Steel

**DOI:** 10.3390/ma15165769

**Published:** 2022-08-21

**Authors:** Hongbin Shang, Zhiyuan Lin, Hongli Gao, Xiaofeng Shan, Jingsong Zhan

**Affiliations:** Key Laboratory of Special Equipment Manufacturing and Advanced Processing Technology of the Ministry of Education, Zhejiang University of Technology, Hangzhou 310023, China

**Keywords:** physical short crack, Q&P steel, crack closure, Micro-DIC, micro-crack morphology

## Abstract

In this paper, a novel dual microscopic fatigue-crack and tip-deformation-fields measurement method based on a hybrid image-processing technique is proposed that was used to research the physical short fatigue crack (SFC) closure effect and the evolution law of the tip deformation fields of Quenching–Partitioning (Q&P) steel during the crack-closure process. The measurement problems are solved, such as the small SFC tip region, large deformation gradient, and strong material anisotropy. Microscopic crack and speckle images are acquired simultaneously on both sides of a compact tensile (CT) specimen of Q&P steel by dual microscopic cameras. A digital image processing (DIP) method is used to identify crack-growth morphology and measure crack length in Q&P steel, and the SFC growth rates are analyzed under different stress ratios. Microscopic digital image correlation (Micro-DIC) is used to analyze displacement fields at the crack tip of SFC and, combined with virtual extensometer technology, analyze the evolution law of crack closure and the evolution of crack-growing morphologies during the closure process under different lengths and stress ratios. Accordingly, the evolution of strain fields at the crack tip in one load cycle for different crack lengths and stress ratios during the SFC closure process is analyzed. The results show that the stress ratio affects the crack-closure behavior and crack growth rate of Q&P steel in the physical SFC crack-growing stage. The crack-closure effect has an obvious influence on the evolution process of displacement and strain fields at the crack tip. The evolution of short-fatigue-crack-tip morphology and strain field of Q&P steel conforms to the crack-closure law. The research results provide experimental and theoretical support for the further study of the SFC growth mechanism and fatigue life prediction of Q&P steel.

## 1. Introduction

Energy saving, safety, and environmental protection are the core issues in the development of the modern automobile industry. Advanced high-strength steel (AHSS) has become a new material for automobile manufacturing in recent years because of its high strength and high toughness [1,2]. Quenching–Partitioning (Q&P) steel is the third-generation AHSS with transformation-induced plasticity (TRIP) properties. It not only meets these requirements, but also achieves a good match between cost and performance. Therefore, it is a representative new material of steel for automobile manufacturing in the future [3,4,5].

The main parts of the automobile are constantly in dynamic service state under the excitation of alternating loads during operation. Its dynamic characteristics which directly determine the service life of the automobile cannot be fully described simply by using the static tensile properties of the material. Therefore, in the design of AHSS used for thin-walled lightweight structures of automobile bodies, not only the fatigue life limit analysis of the material is required, but also the crack initiation and growth characteristics under alternating loads [6,7].

Fatigue crack growth (FCG) includes three stages: crack initiation, SFC growth, and long crack growth. SFC is divided into microstructure short crack (MSC) and physical short crack (PSC). A short crack whose crack size is equivalent to the characteristic size of the material microstructure is called MSC. The crack size obviously exceeds the characteristic size of the microstructure, and with a length of 1–2 mm, it is called a PSC. Under the same nominal stress intensity factor, the SFC growth rate is much higher than that of a long crack [8], and the short crack can still grow under the long-crack growth threshold, the phenomenon cannot be explained by the long-crack growth model. Since the 1980s, numerous investigations have gradually shifted from the propagation behavior of long cracks (≥2 mm) to the propagation behavior of small cracks (10 μm–2 mm). Nowadays, the SFC growth behavior has become a major research hotspot in the field of fatigue fracture [9,10]. The propagation behavior of short crack is mainly determined by the microstructure of the material, the applied load, the plastic zone near the crack tip region, and the crack-closure behavior. For PSC, the crack propagation is not affected by the microstructure of the material; in addition to the applied load, the crack-tip closure effect and the deformation fields distribution are the main factors affecting the fatigue-crack propagation [11,12,13]. Because crack closure affects the distribution of the stress–strain fields near the crack tip, the range of the stress intensity factor is reduced; thereby, the crack’s growth rate becomes slow [14]. In this paper, a hybrid digital-image-processing method based on Micro-DIC is proposed to research the effect of physical-short-crack closure in Q&P steel, the growing morphology of physical short crack, and the evolution law of tip-deformation fields during the crack-closure process under different stress ratios and different lengths.

Measuring the deformation fields of the small-crack tip region in Q&P steel faces some challenges, such as a complex microstructure with fine grain, stress and strain distribution with large variation gradient, rapid evolution over time, and high measurement accuracy; overall, Micro-DIC is the most suitable method. DIC has some advantages over other global photometric methods, such as in situ X-ray diffraction [15,16], Holographic interferometry [17,18], Moiré interferometry [19,20], and Laser speckle photography [21,22], including a high measurement accuracy, a simple experimental setup, and the need for less specimen preparation, and better environmental adaptability. Moreover, using DIC helps obtain instantaneous full-field 2D and 3D displacement and strain-field data and meet the requirements of in situ measurement and observation of the multi-scale fatigue-crack deformation and propagation evolution process at the fatigue crack tip. DIC, including Micro-DIC, has often been used to study the deformation behavior, fatigue life, and propagation characteristics of fatigue crack tip in the past ten years. Gao et al. [23] proposed a macroscopic hybrid image-processing method, using the DIP method to determine the macroscopic crack location and path, and using the DIC method to obtain the strain fields and plastic zone size in the macroscopic crack tip region. Gonzáles et al. [24] used the macroscopic DIC technique to analyze the discontinuity of crack closure after a single tensile overload under a constant ΔK load. Hu et al. [25] used Micro-DIC to study laser-strengthening-induced crack closure and its effect on fatigue life extension of aluminum alloys. Chen et al. [26] used a microscopic camera system with a spatial resolution of 2 µm/pixel combined with DIC to obtain the crack-tip-area displacement, strain field, and crack-opening displacement during variable amplitude loading and studied the overload hysteresis behavior of crack propagation.

In this paper, a novel dual microscopic fatigue crack and tip deformation fields measurement method–based hybrid image-processing technique is proposed that solves the measurement problems of small SFC tip regions, large deformation gradients, and the strong material anisotropy of Q&P steel. The spatial resolution of the cameras is 0.338 μm/pixel and 1.032 μm/pixel, respectively. Microscopic crack and speckle images are acquired simultaneously on both sides of a CT by dual microscopic cameras, and microscopic DIC technology is used and combined with DIP technology to process the acquired images, as it can accurately and synchronously measure the crack-tip deformation fields and crack morphology during the crack-closure process.

In recent years, several studies have analyzed the Q&P fatigue-crack growth characteristics and achieved fruitful results. Hockauf et al. [27] studied the effects of retained austenite content on the propagation law of Q&P steel in the steady-state crack-growth stage of Paris and the threshold value of crack growth, ΔKth, and they published the first article on the Q&P steel fatigue-crack-propagation mechanism. Golling et al. [28] studied the effect of the strain loading rate on the fracture toughness of Q&P steel, using the Micro-DIC method to measure the local strain field at the crack tip region of Q&P steel specimens with prefabricated cracks under different strain-loading rates. Song et al. [7] studied the fatigue life, fatigue-crack growth characteristics, and fatigue-crack microscopic morphological characteristics of a Q&P steel; they also studied the influence of residual austenite content and stability. In addition, there is no report on the research about the deformation fields of SFC tip, microscopic morphology of short crack, crack-closure effect, short-crack propagation law, and mechanism of Q&P steel.

In this paper, a novel dual microscopic fatigue-crack and tip-deformation-field measurement method based on DIC, DIP, and virtual extensometer technology is proposed that solves multiple measurement problems. The effect of crack closure on the evolution law of SFC and near crack-tip-region deformation fields in Q&P steel was studied for the first time. In the fatigue test, 50 speckle images and 50 microscopic crack images under different load values were collected simultaneously in one load cycle to record the closing process of the crack, that is, the process of the crack from closing to opening, and then from opening to closing during a full loading–unloading–loading cycle. The evolution law of fatigue short crack closure was analyzed. In particular, the images of different load levels P/maximum load Pmax were selected in a cycle to analyze the evolution law of SFC morphology and crack-tip-region deformation fields. Finally, some conclusions are given, and future work is discussed based on the current investigation.

## 2. Materials and Methods

### 2.1. Overview of the Method

**Figure 1:**
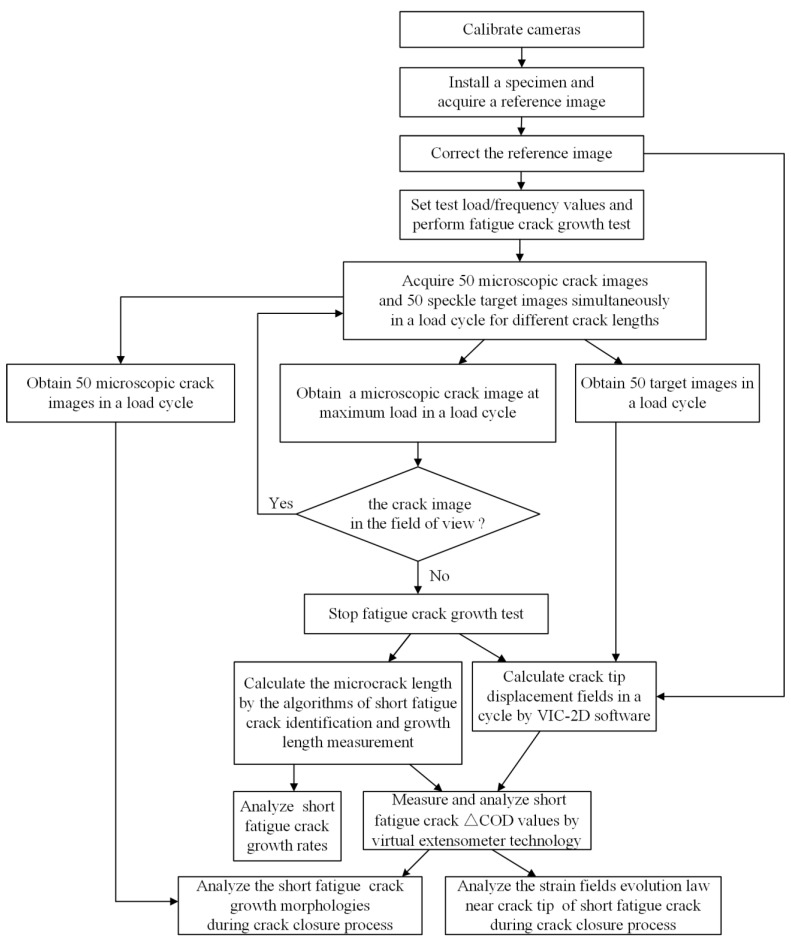
Overview of the proposed method.

The flowchart of the overall method proposed in this paper is shown in Figure 1. Preparations before the FCG test included adjusting systems, calibrating cameras, and preparing CT specimens with microscopic speckles. The microscopic cameras were calibrated by using Zhang’s calibration method [29]. Two calibration boards with different specifications were made. Microscopic cameras collect images from 6 different orientations separately. Distortion coefficients of the cameras are calculated by using the spatial locations of these images. One side of the specimen is polished to a smooth surface with diffuse effect, and the other side is covered with microscopic speckles. The specimen is installed on the hydraulic servo fatigue tester, and a microscope speckle image of crack tip without load is acquired and corrected to be used as a reference image for DIC. The FCG test is performed after setting the load and frequency values. A total of 50 microscopic crack images and 50 speckle target images are acquired simultaneously in a load cycle at different crack lengths by dual microscopic cameras, and the acquired images are in the defined position in the computer. The microscopic crack image under the maximum load is observed; when the crack image is in the field of view, the experiment will continue. Conversely, the test is stopped when the crack length exceeds the field of view. The crack length is calculated and the SFC growth rate is analyzed by using SFC identification and length measurement algorithms to process the microscopic crack images at maximum force value. Speckle target images are corrected, and displacement fields of the SFC tip are calculated by using VIC-2D software. The crack-opening displacement (COD) is measured by using the virtual extensometer technique to determine the SFC crack-closure behavior and morphologies during the crack-closure process. The evolution law of strain fields near the crack tip region of SFC is obtained during the crack-closure process, according to the displacement fields of SFC and the evolution law of crack-closure effect.

### 2.2. Materials and Specimens

**Figure 2:**
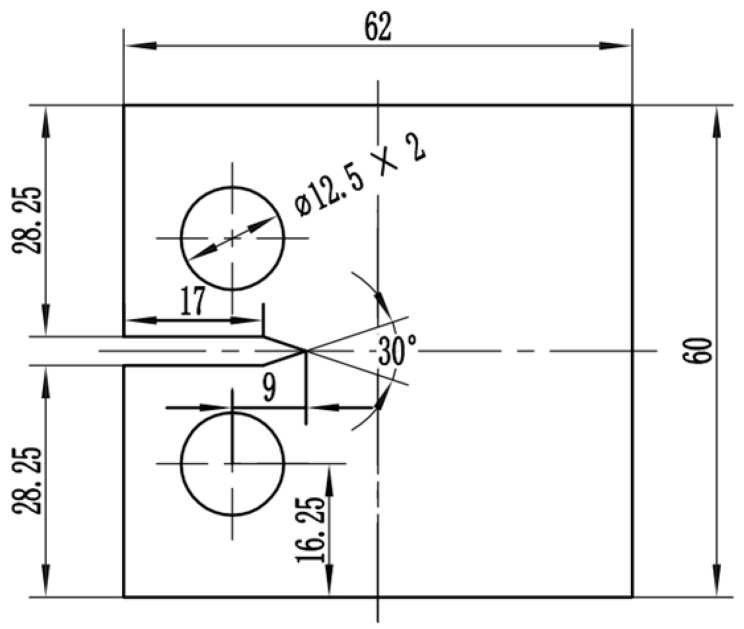
Geometric dimensions of CT specimen.

The material used in this investigation is Q&P980 high-strength steel. The mechanical properties under room temperature obtained from material tensile test are listed in Table 1. The chemical composition of base metal in wt.% includes Cr 15.6, Mo 0.63, O 0.55, Nd 0.25, Sn 0.11, C 0.11, and Fe is the balance. An average grain size of the material was measured to be approximately 4.5 µm. All the CT specimens were machined from the same steel plate. The geometric dimensions of CT specimens are 62 mm (length) × 60 mm (width) × 1.4 mm (thickness), as shown in Figure 2. The side of the specimen is polished to a smooth surface with a diffuse effect, making it convenient to observe and identify cracks. Microscopic speckles were prepared on the other side of the sample for microscopic DIC analysis.

### 2.3. System Components

**Figure 3:**
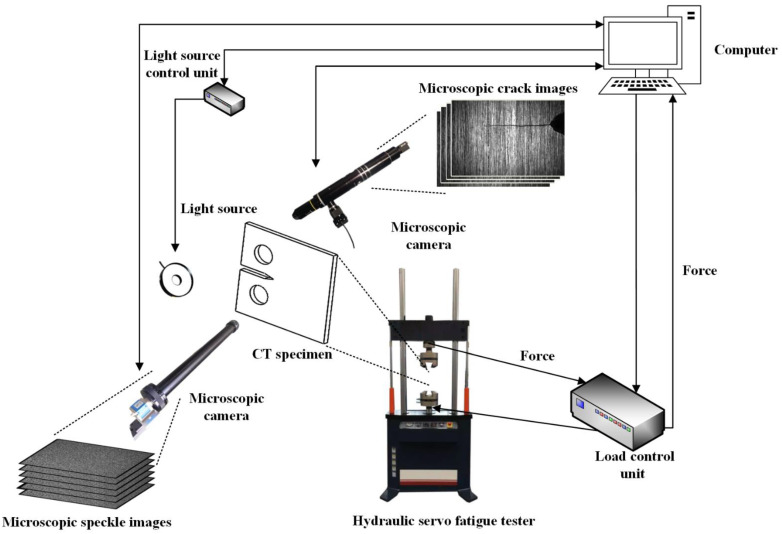
Schematic representation of the system components.

As shown in Figure 3, the system components are a hydraulic servo fatigue tester (PWS-50), CT specimen, load control unit, dual microscopic cameras, light source, light source control unit, and computer with fatigue test control software; microscopic-image-acquisition software; digital-image-processing software, including VIC-2D and DIP short-fatigue-crack identification and measurement software. During the FCG test, the load sensor transmits the force applied on the specimen to the load control unit, which converts the analog force signal into a digital signal and feeds it back to the computer to realize FCG, microscopic-image acquisition, and digital-image processing.

The BFS-U3-200S6M-C camera with a resolution of 5472×3648 pixel, produced by FLIR company, and the Resolv4K lens, which was produced by NAVITAR company, realize microscopic-crack-image acquisition. The acA4112–30 µm camera with a resolution of 4096×3000 pixel, produced by BASLER, and the Rodagon 5.6/105 metal lens, which was produced by LINOS company, realize microscopic-speckle-image acquisition. The main hardware parameters of cameras and lenses are shown in Table 2.

The depth of field of the Resolv4K lens is so small that the working distance between the lens and the specimen is only 10 mm, and the original fixtures cannot meet the testing requirements. Therefore, a novel microscopic fixture is designed

### 2.4. Microscopic Fatigue Crack and Speckle Images’ Acquisition

**Figure 4:**
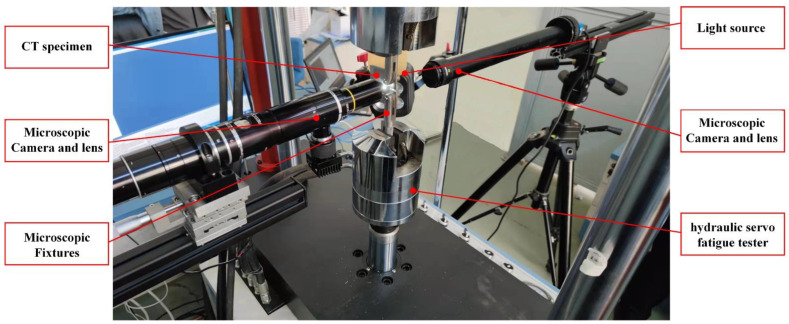
Labeled photo of the experimental platform.

A labeled photo of the experimental platform is shown in Figure 4. The CT specimen is installed on the hydraulic servo fatigue tester. Dual microscope cameras are installed on both sides of the specimen. The morphology and length of microscope cracks are acquired on one side of the specimen. The working distance between the lens and the specimen is 10 mm, and the field of view is 2 mm×1.3 mm. The transverse field of view is slightly larger than the length of the SFC. The spatial resolution of the corrected image is 0.338 μm/pixel. The other side is used for microscope speckle images’ acquisition. Since the microscope speckle images need Micro-DIC analysis, the transverse field of view is larger than the length of SFC. The distance from the lens to the specimen is 110 mm, and the field of view is 4 mm ×2.1 mm. The spatial resolution of the corrected image is 1.032 μm/pixel. A speckle image of the specimen is acquired and corrected as the reference image.

The fracture toughness,$\;K_{c}$, of the CT specimen under quasi-static loading is $45\;\mathrm{MPa}\sqrt{m}$. In this paper, the sinusoidal alternating load is Fmax =3.0 kN, and the initial maximum stress intensity factor is $\mathrm{Kmax}=6.38\;\mathrm{MPa}\sqrt{m}$ under the load. According to theoretical estimation and previous experimental results, the steady-state growth of fatigue cracks can be guaranteed when the crack-growth length is less than 10 mm.

Table 3 shows the FCG test parameters. After setting the test load and frequency on the fatigue-test-control software, the FCG test is performed. When the short crack grows to different lengths, image acquisition begins. In the acquisition process, the vibration frequency is reduced to 0.01 Hz. The image acquisition software receives the force signal from the load control unit. When the force value is equal to the set acquisition load value, the dual cameras are triggered synchronously to acquire 50 microscopic crack images and 50 speckle target images within a load cycle.

## 3. SFC Tip Closure and Deformation Fields Measurement Method Based on Micro-DIC

### 3.1. Microscopic Speckles’ Preparation

**Figure 5:**
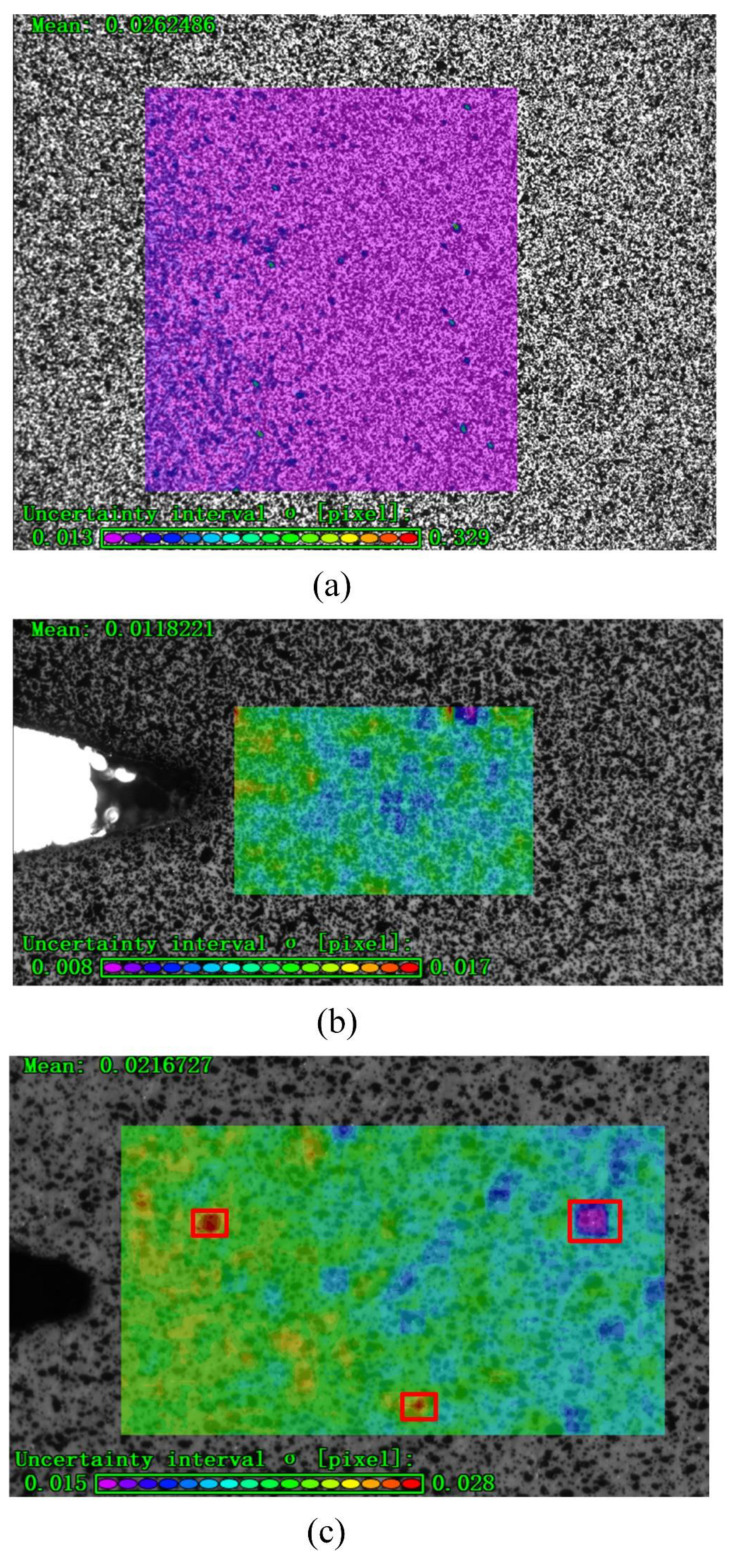
Confidence assessment: (**a**) the confidence of ROI in macroscopic speckle image, (**b**) the confidence of ROI in microscopic speckle image, and (**c**) microscopic speckles are re-prepared.

For DIC analysis, the confidence of region of interest (ROI) in the speckle image is required to be close to the reference value of 0.01 given in the VIC-2D software manual. As shown in Figure 5a, the confidence of ROI in the macroscopic speckle image is 0.024, which cannot meet the test requirements. Therefore, microscopic speckles are prepared. As shown in Figure 5b, the confidence of ROI in the microscopic speckle image is around 0.01, which meets the experimental requirements. There are three obvious too-high or too-low confidence points in the center of the ROI, all of which are highlighted with a red rectangle, and the microscopic speckles need to be re-prepared, as shown in Figure 5c.

In this paper, a professional airbrush is used to prepare microscopic speckles. The diluted white paint is added to the airbrush, so that the sprayed white particles evenly fall on the surface of the CT specimen. In the same way, we spray black paint. Prepared microscopic speckles do not slip, fall off, and discolor with the material surface under loading conditions, and we try to ensure uniformity and isotropy. The prepared speckle images are acquired for confidence assessment. If the confidence requirements are not met, the surface of the CT specimen can be lightly polished with metallographic sandpaper to remove the black and white paint and then be re-prepared.

### 3.2. Displacement and Strain Fields Calculation

To calculate displacement fields of the short crack tip, two microscope speckle images are used. One is the reference image selected before deformation, and the other is the image after deformation. The ROI (2000×1000 pixel) is drawn near the crack tip of the reference image, and the region that is not selected is taken as the irrelevant region. The ROI is divided into several 125×125 pixel regions as subsets, and the subset size controls the area of the image that is used to track the displacement between images. The cross-correlation function zero-mean normalized cross-correlation function [30] is used for similarity matching to find a central target subset at $\;P^{\prime }\left(x_{0}^{\prime },y_{0}^{\prime }\right)$, which is a deformation of the centered reference subset at $P\left(x_{0},y_{0}\right)$. The function is used to calculate the displacement of any point $\;Q^{\prime }\left(x_{i}^{\prime },y_{i}^{\prime }\right)\;$ in the target subset relative to the corresponding point $Q\left(x_{i},y_{i}\right)\;$ in the reference subset to obtain the displacement fields of the short crack tip, as shown in (1):

(1) $$\begin{array}{c} x_{i}^{\prime }=x_{i}+\mu +\mu_{x}\Delta x+\mu_{y}\Delta y\\ y_{i}^{\prime }=y_{i}+\nu +\nu_{x}\Delta x+\nu_{y}\Delta y \end{array}$$

where *μ* represents the displacement of the center of the reference subset in the *x*-axis direction,$\;\mu =x_{0}\prime -x_{0}$; *ν* indicates the displacement of the reference subcenter in the *y*-axis direction; $\mu_{x}$, $\;\mu_{y}$, $\nu_{x}$, and $\nu_{y}$ are the partial derivatives of the central displacement of the reference subset in the *x*-axis and *y*-axis direction, indicating the displacement gradient of the reference subset; and $\Delta x=x_{i}-x_{0}$ and $\Delta y=y_{i}-y_{0}$ represent the distance from point $Q\left(x_{i},y_{i}\right)$ to the center $P\left(x_{0},y_{0}\right)$ of the reference subset.

Then strain fields can be directly calculated by differentiation of displacement fields’ data. In order to reduce noise, the strain is described by the Lagrange strain tensor, and the local least squares transformation technique is used to solve the strain [23]. In this paper, the software VIC-2D is used to calculate the displacement and strain fields at the crack tip of CT specimen.

### 3.3. Fatigue-Crack-Closure Measurement

**Figure 6:**
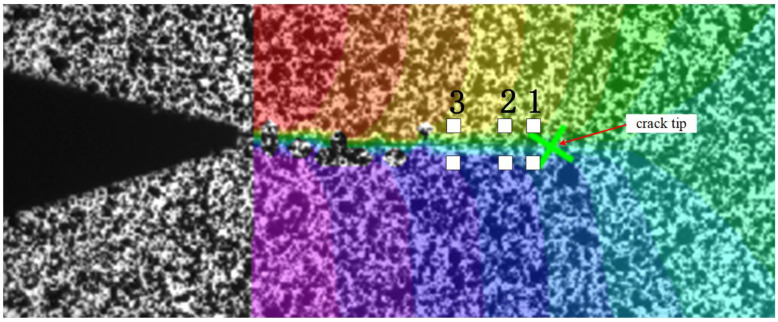
Virtual extensometers’ measuring point arrangement. (The three numbers represent three pairs of virtual extensometers).

Whether the crack is closed or not is generally characterized by measuring the crack-opening displacement within a fatigue cycle. The crack-opening displacement of crack tip cannot be accurately measured by using the traditional compliance method [26]. Nowell et al. [31] and Yusof et al. [32] also indicated that using virtual extensometers to measure COD values near the crack-tip point can reflect the closure response well. In this paper, the virtual extensometer technique is used to measure fatigue-crack-opening displacement [26,33], Δ*COD*, at various distances behind the crack-tip by subtracting vertical displacements of the top flank and bottom flank of the crack mouth:

(2) $$△COD(x)=u_{y}^{\mathrm{top}}-u_{y}^{bot}$$

where the Δ*COD* varies as a function of distance behind the crack-tip; *y* and *x* are the vertical and horizontal direction, respectively; and $u_{y\;}$ is the vertical displacement. Three pairs of virtual extensometers measurement points are arranged at 20 μm, 40 μm, and 80 μm behind the crack tip of the displacement fields image, as shown in Figure 6. According to the crack-opening displacement under different loads in a load cycle, the P/Pmax−ΔCOD curve can be obtained to further analyze the effect of crack closure on FCG, where *P* is the current load, and Pmax is the maximum load.

## 4. SFC-Identification and Length-Measurement Algorithms Based on DIP

SFC-identification and length-measurement algorithms include the pre-crack’s notch-region-matching algorithm, SFC’s initiation-point-detection algorithm, and SFC’s growth-path-detection algorithm.

### 4.1. Pre-Crack’s Notch-Region-Matching Algorithm

In this paper, the normalized cross-correlation (NCC) function is selected to locate the prefabricated crack region, as shown in in the following:

(3) $$NCC\left(r,c\right)=\frac{1}{n}\underset{u,v\in R}{\sum }\frac{t\left(u,v\right)-m_{t}}{\sqrt{s_{t}^{2}}}\cdot \frac{i\left(r+u,c+v\right)-m_{i}\left(r,c\right)}{\sqrt{s_{i}^{2}\left(r,c\right)}},\;$$

where (r,c)  is the offset of the template image window on the target image; n is the total number of pixels in the template image; *R* is the region of template image; t(u,v) is the gray value of the pixel point in the template image: $m_{t}$ is the average gray level of the template image: $s_{t}^{2}\;$ is the grayscale variance of the template image; i(r+u,c+v)  is the gray value of the pixel at coordinate (r+u,c+v) in the target image; $m_{i}\left(r,c\right)\;$ is the grayscale mean of the template image window region with offset (r,c) on the target image; and $s_{i}^{2}\left(r,c\right)$ is the grayscale variance of the template image window region with offset (r,c)  on the target image.

At the microscopic scale, the processing difference of the pre-crack notch region is magnified. In order to improve the matching accuracy, a separate template is designed for each specimen.

### 4.2. Short Fatigue Crack’s Initiation-Point-Detection Algorithm

Preprocessing and focus detection are performed separately to obtain the SFC initiation point. For sharper edges and stronger contrast, image enhancement is performed on the pre-cracked region. A window of size M×N (pixels) is selected to slide on the image, and the gray value of the center point of the window is replaced with the result of (4), where *res* is the resulting gray value, *round*() is the rounding function, *orig* is the gray value of the center point of the window, *mean* is the average gray value of the window, and *Factor* is the contrast enhancement factor. For the edge area that cannot meet the window size requirements, the mirroring method can be used to fill in the pixel-free area:

(4)  res=round((orig−mean)∗Factor)+orig  

After preprocessing, the image is binarized. Then the Harris corner extraction method is used to detect corners, as shown in (5), where matrix M(x,y)  is called the Hessian matrix, t(x,y)  is the window centered at point (x,y), G  is the Gaussian weighting function, and $\;I_{x\;}\mathrm{and}\;I_{y}\;$ are the first-order partial derivatives of *y*.

(5) $$M\left(x,y\right)=\sum_{t\left(x,y\right)}G*\left[\begin{array}{cc} I_{x}^{2} & I_{x}I_{y}\\ I_{x}I_{y} & I_{y}^{2} \end{array}\right]\;$$

The corner response function, *R*, is used to judge whether the pixel is a corner, as shown in (6), where *DetM* represents the determinant of the matrix *M*, *Alpha* indicates weight, and *TraceM* represents the matrix *M* trace.

(6) $$\;\;\;R=DetM-Alpha*{\left(TraceM\right)}^{2}\;\;\;\;\;\;\;$$

### 4.3. Short Fatigue Crack’s Growth-Path-Detection Algorithm

In this algorithm, the gray mean square error method (GMSE) is used to identify the crack area, and Gaussian filtering, adaptive threshold filtering, and crack skeleton extraction are used to detect the crack growth path.

Twelve groups of subsets are distributed horizontally from the initiation point, and each group of subsets includes upper and lower subsets with a width and a length of 400 pixels. The size of the subsets is determined with reference to the resolution of the camera and multiple tests, and the crack region is identified by the GMSE method.

The main body of microscopic short cracks is a thick black continuous region with fine bifurcations, and Gaussian filtering is selected for preprocessing. Adaptive threshold segmentation is used to process crack images. The threshold of each pixel in the adaptive threshold algorithm is determined by the Gaussian weighted mean t(x, y)  in the N × N pixel’s neighborhood, with itself as the center, as shown in (7):

(7) $$t\left(x,y\right)=\overset{a}{\underset{r=-a}{\sum }}\overset{a}{\underset{l=-a}{\sum }}w\left(l,r\right)\cdot f\left(x+l,y+r\right)\;$$

where  a=(N−1)/2,  w (l,r)  is the Gaussian weighted mean function, and  f(x+l,y+r) is the pixel whose coordinates are (x+l,y+r)  in the image Point gray value.

The final threshold segmentation function of image *I (x, y)* is shown in (8):

(8) $$dst\left(x,y\right)=\{\begin{array}{c} 255\\ 0 \end{array}\begin{array}{c} ,\;I\left(x,y\right)\leq t\left(x,y\right)\\ ,\;I\left(x,y\right)>t\left(x,y\right) \end{array}$$

The small regions connected to the target crack after threshold segmentation are removed by opening arithmetic, and the reconstructed crack skeleton extraction is used for extraction. The process of reconstructing the skeleton includes the following steps: First, the original skeleton is transformed into a line segment containing only eight neighborhoods. Then line segment endpoints and intersections are marked. The new line segment is generated as an endpoint and an intersection with the (9) feature, where 0 is the background,1 is the foreground, and 2 is the intersection:

(9) $$\;\left[\begin{array}{ccc} 1 & 0 & 1\\ 0 & 2 & 0\\ 0 & 0 & 1 \end{array}\right]\;\;\;\left[\begin{array}{ccc} 1 & 0 & 1\\ 0 & 2 & 0\\ 1 & 0 & 1 \end{array}\right]\;\;\;\left[\begin{array}{ccc} 1 & 0 & 0\\ 0 & 2 & 1\\ 0 & 1 & 0 \end{array}\right]\;\;\;\left[\begin{array}{ccc} 1 & 0 & 0\\ 0 & 2 & 1\\ 1 & 0 & 0 \end{array}\right]\;\;\;\left[\begin{array}{ccc} 0 & 1 & 0\\ 0 & 2 & 1\\ 0 & 1 & 0 \end{array}\right]\;\;\;\left[\begin{array}{ccc} 0 & 1 & 0\\ 1 & 2 & 1\\ 0 & 1 & 0 \end{array}\right]$$

After crack skeleton extraction, some false crack skeletons can be found. Therefore, all skeletons are checked with distance and slope, and the false skeletons are removed. According to the decision of the camera resolution and the location of the bifurcation cracks generated by multiple tests, the search distance is set to 50 pixels, and |k1 – k2| ≤ 0.15, where *k*1 is the slope of initial crack, and *k*2 is the slope of crack bifurcation.

## 5. Results and Discussion

### 5.1. Short Fatigue Crack’s Length Measurement and Growth Law in Q&P Steel

**Figure 7:**
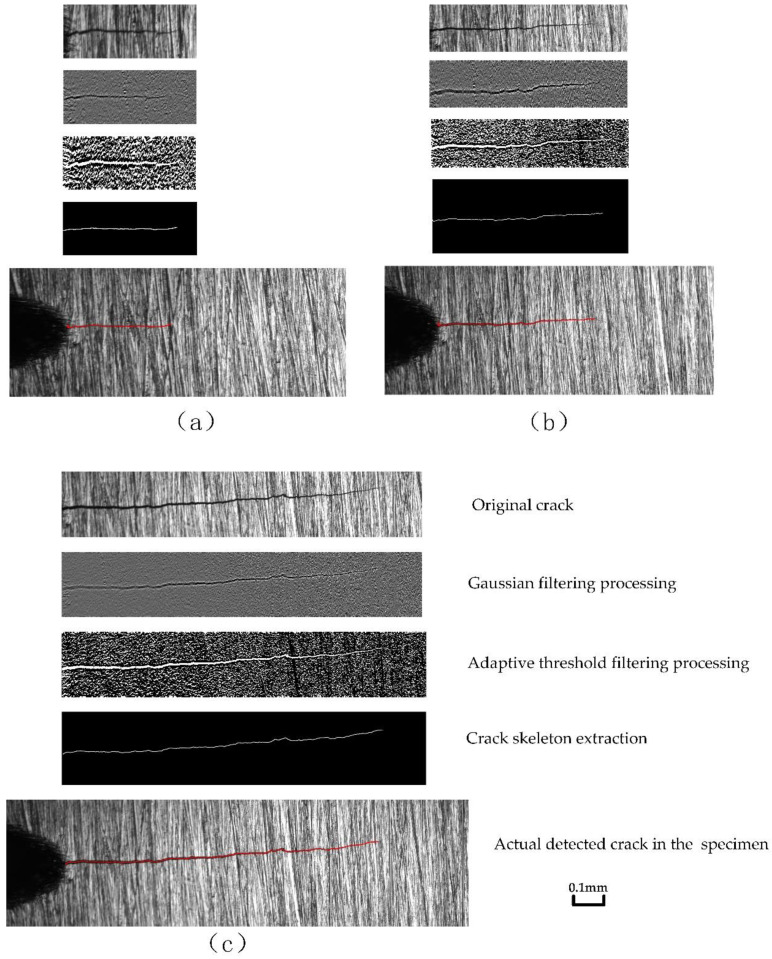
Result images in the process of crack extraction by the proposed method, with different cracks length and a stress ratio of 0.4: (**a**) crack length of 0.314 mm, (**b**) crack length of 0.859 mm, and (**c**) crack length of 1.507 mm. (The red line represents the detected crack.).

According to the microscopic crack image at the maximum force value, the crack length is calculated by using SFC identification and length-measurement algorithms proposed in Section 4. During the process of fatigue crack growth, the result images were calculated by the proposed method at the stress ratio of 0.4, as shown in Figure 7, which includes three sets of result images. Each set of result images includes five subfigures, which are original crack, Gaussian filtering processing, adaptive threshold filtering processing, crack skeleton extraction, and actual detected crack in the specimen.

In order to verify the accuracy of the crack length, the results of crack length obtained by the algorithms were compared with the measurement results obtained by using an optical microscope with the accuracy of 0.001. The specific process is shown in Reference [23]. The comparison results of the crack length are shown in Table 4.

**Figure 8:**
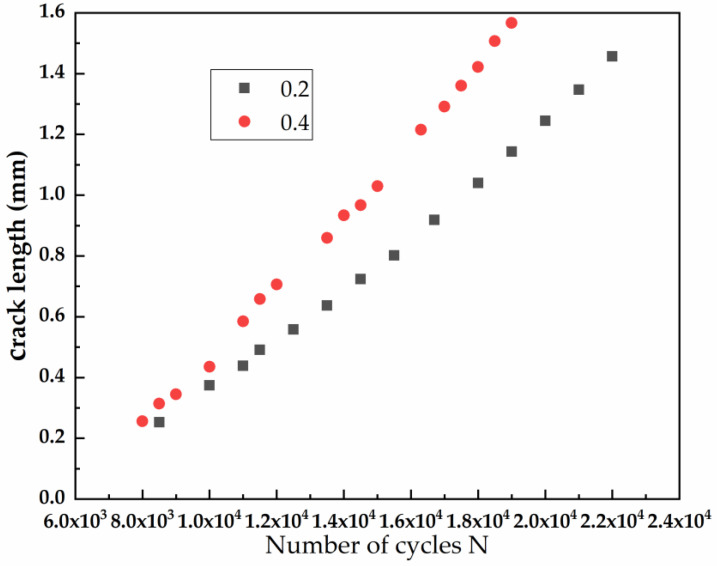
The FCG rate under two stress ratios.

Figure 8 shows the FCG rates under two stress ratios. During short-crack growth stage, the FCG rates increase slightly with the increase of cycle number, and the larger the stress ratio, the faster the FCG rate. The difference of FCG rate between two stress ratios also increases gradually with the number of cycles.

### 5.2. Short Fatigue Crack Tip’s Displacement Fields and Crack-Closure Evolution in Q&P Steel

The Micro-DIC technique was performed on the crack area of about 2 mm × 1 mm in the acquired microscopic speckle images. Since the deformation of the CT specimen is mainly concentrated in the vertical direction, the vertical displacement fields were mainly analyzed.

**Figure 9:**
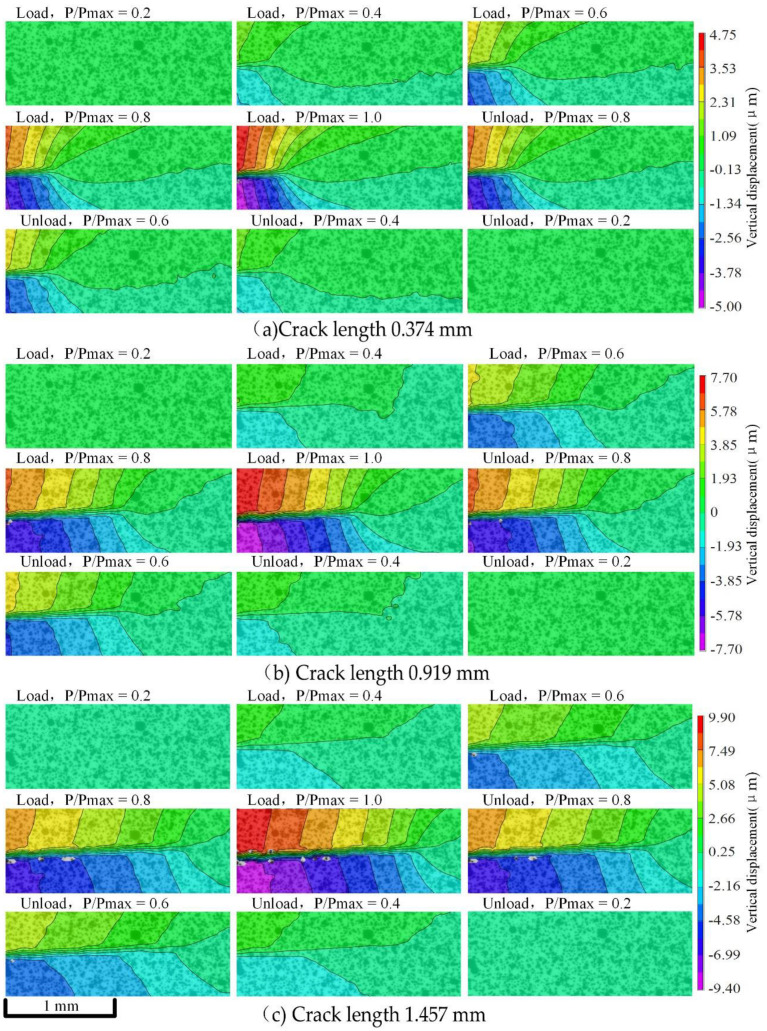
The vertical displacement fields in a load cycle at different crack lengths when the stress ratio is 0.2.

**Figure 10:**
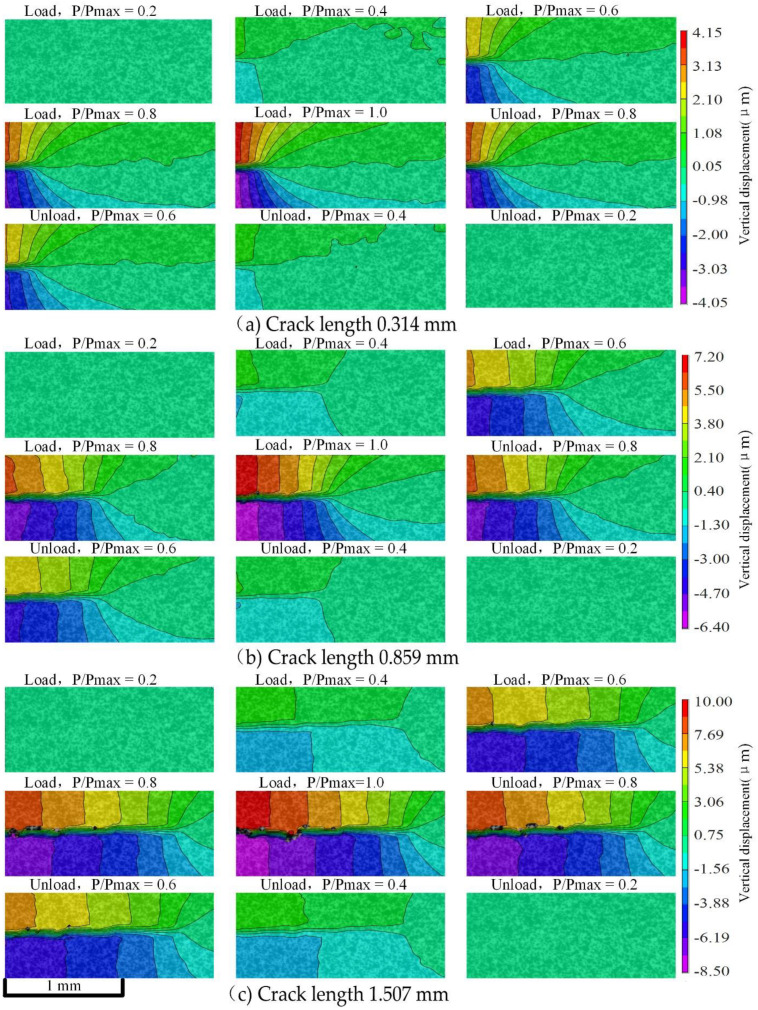
The vertical displacement fields in a load cycle at different crack lengths when the stress ratio is 0.4.

The vertical displacement field results at two different stress ratios are shown in Figure 9 and Figure 10, which include three sets of images at different crack lengths, respectively. Each set of images includes nine resulting images at a different P/Pmax (P is the current load, and Pmax is the maximum load) in one loading cycle. At the same stress ratio, vertical displacement increases with the growth of the crack length. Vertical displacement in a load cycle is basically symmetrical during loading and unloading. When the load is near P/Pmax =0.8, the vertical displacement changes greatly. When the two stress ratios are close, the increase of vertical displacement is highly correlated with the crack length and is less correlated with the stress ratio.

**Figure 11:**
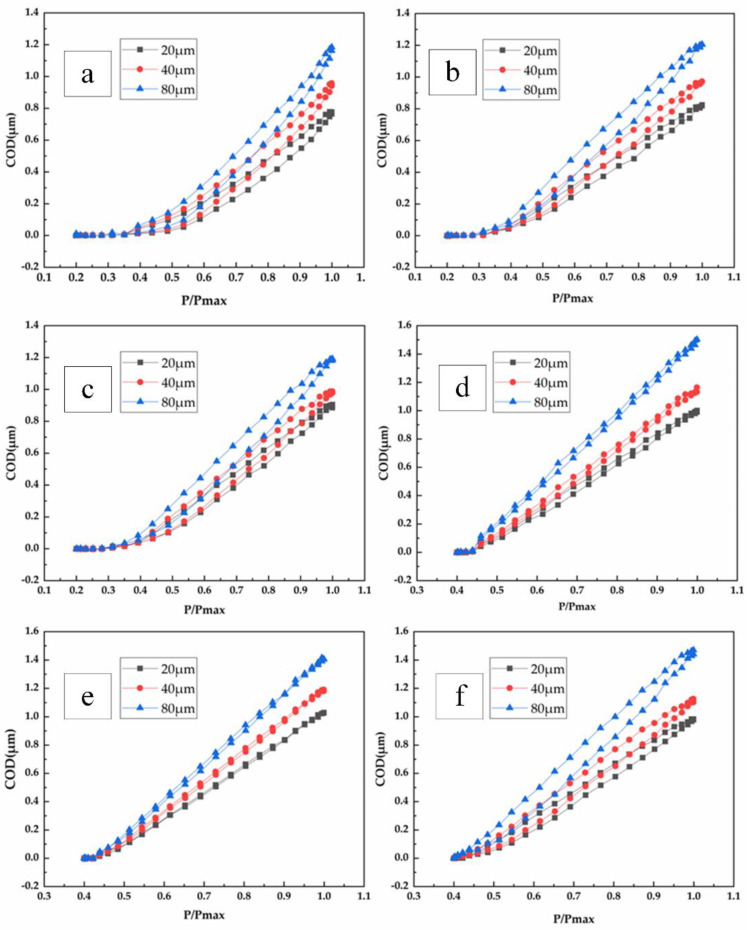
P/Pmax-ΔCOD curves of different lengths at stress ratio of 0.2: (**a**) crack length 0.374 mm, (**b**) crack length 0.919 mm, and (**c**) Crack length 1.457 mm. P/Pmax-ΔCOD curves of different lengths at stress ratio of 0.4: (**d**) crack length 0.314 mm, (**e**) crack length 0.859 mm, and (**f**) crack length 1.507 mm.

In Section 3.3, according to the crack-opening displacement under different loads in a load cycle, the P/Pmax-ΔCOD curve was obtained. The P/Pmax-ΔCOD curves at different crack lengths of stress ratios of 0.2 and 0.4 are shown in Figure 11. As shown in Figure 11a, when  P/Pmax =0.4, the state of the crack tip changes from a closed state to an open state. As the load increases gradually, the crack-closure behavior weakens until the crack opens completely. As shown in Figure 11c, after P/Pmax=0.4, the crack-opening speed increases with the increase of load. As shown in Figure 11d, when P/Pmax=0.43, the state of the crack tip changes from closing to opening. To sum up, under the same stress ratio, the longer the crack is, the weaker the crack-closure behavior is. At the same crack length, the smaller the stress ratio is, the stronger the crack-closure behavior is.

In the same fatigue cycle, the maximum opening displacement at 20 μm, 40 μm, and 80 μm behind the crack tip increases slightly with the crack growth. In the vicinity of P/Pmax=0.5, the crack closure at 20 μm and 40 μm behind the crack tip is basically the same, and the crack at 80 μm opens faster. Under some cases, the crack cannot be completely closed due to the rough surface of the crack.

### 5.3. Short Fatigue Crack’s Morphology Evolution during Crack-Closure Process in Q&P Steel

**Figure 12:**
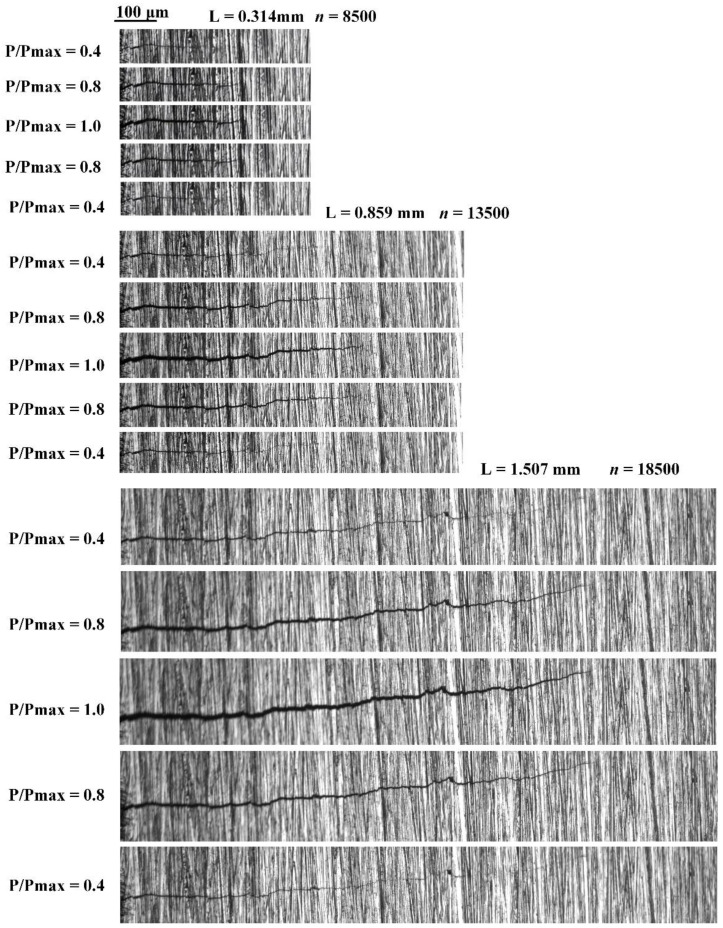
SFC morphology evolution during crack closure with stress ratio of 0.4.

Figure 12 shows the evolution of the SFC morphology during crack closure with different lengths and a stress ratio of 0.4, where P/Pmax represents different load stages. According to the conclusion in Section 5.2, when the stress ratio is 0.4 and the SFC length is 0.859 mm, it will not open until P/Pmax = 0.43. Therefore, under the minimum cyclic load, the crack tip presents a closed state. When the load is from P/Pmax = 0.43 to 1, the crack tip is slightly opened to fully opened, and the closure behavior of short fatigue crack tip is weakened. On the contrary, when unloading, the crack tip exhibits a morphological evolution from fully open to close. The SFC morphologies at different lengths are consistent with the above conditions.

### 5.4. Short Fatigue Crack Tip’s Strain Fields’ Evolution during Crack-Closure Process in Q&P Steel

**Figure 13:**
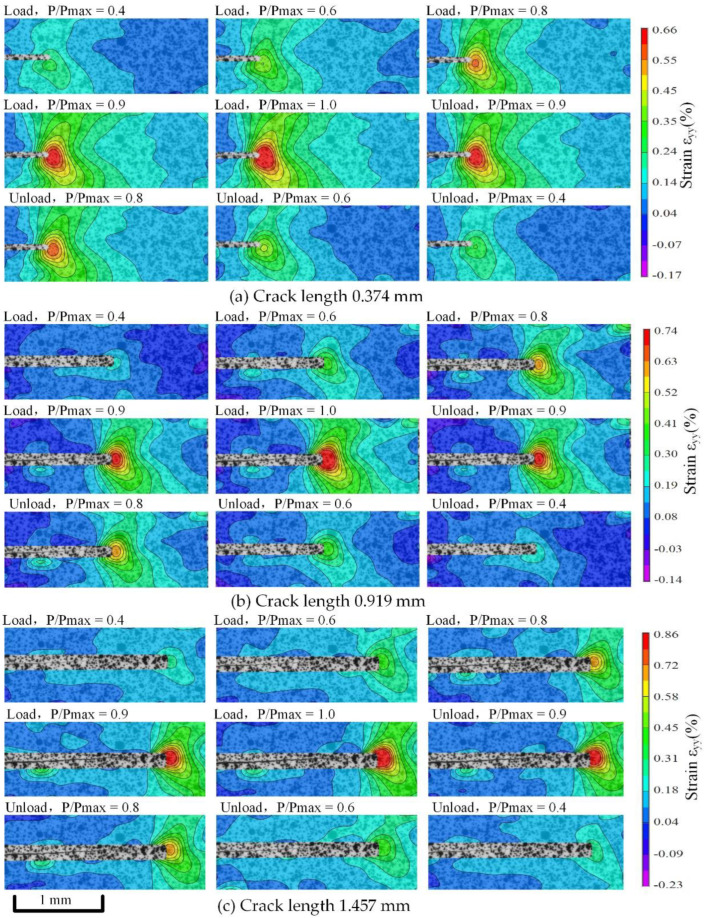
Strain fields’ evolution during crack closure with stress ratios of 0.2.

**Figure 14:**
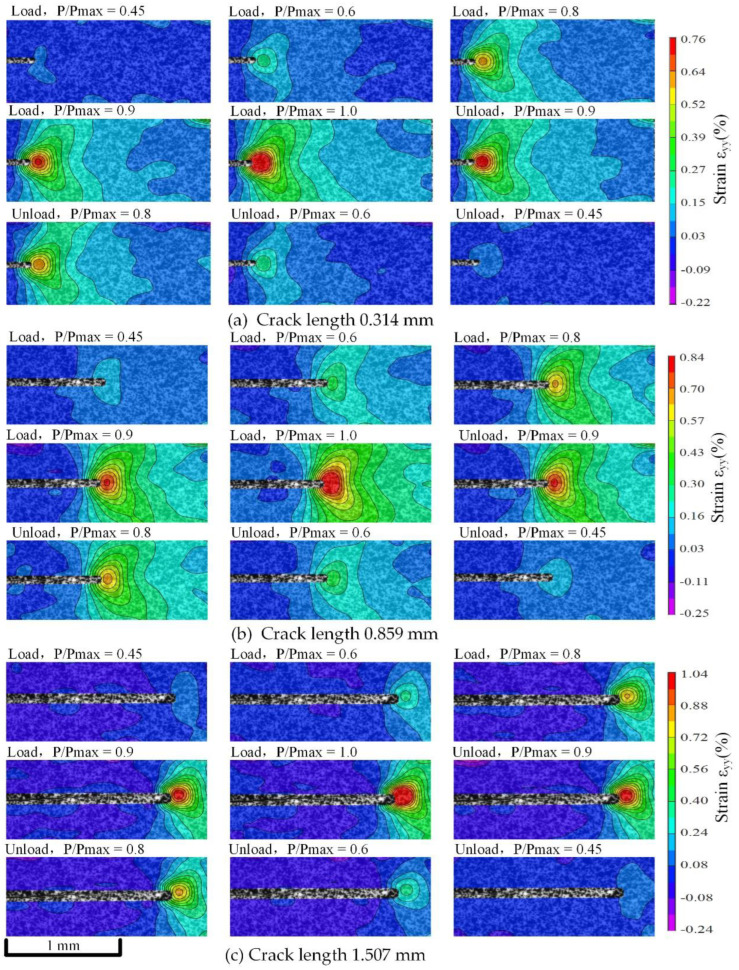
Strain fields’ evolution during crack closure with stress ratios of 0.4.

According to the algorithms in Section 4, the crack length was obtained. Micro-DIC analysis was performed again after deducting the crack area to obtain the real data of stress distribution after SFC growth. Figure 13 and Figure 14 show the evolution of vertical strain fields under each crack length, with stress ratios of 0.2 and 0.4, where P/Pmax represents different load stages. At the same stress ratio, the vertical strain increases with crack growth. In the same fatigue cycle, the vertical strains during loading and unloading are basically symmetrical. It is slightly larger when unloaded, which is related to the inability to fully closing the crack mentioned in Section 5.2.

When the stress ratio is 0.2, the obvious strain is observed at the time of P/Pmax = 0.4; when the stress ratio is 0.4, the obvious strain is observed at the time of P/Pmax = 0.45. This is basically consistent with the load when the crack turns from closing to opening under each stress ratio in Section 5.2, which further proves the correctness of the ΔCOD at the crack tip measured in Section 5.2. It means that the load before the crack tip opens has a small contribution to the increase of the vertical strain at the crack tip. The energy is mainly used to open the crack closure. It shows that crack closure has a great influence on the evolution of vertical strain fields at the crack tip.

## 6. Conclusions

A novel dual microscopic fatigue crack and tip deformation fields measurement method–based hybrid image-processing technique of DIC, DIP, and virtual extensometer technology is proposed in this paper that was used to research the Q&P steel SFC closure effect and the evolution law of the tip deformation fields during the crack-closure process and solve the measurement problems, such as small SFC tip region, large deformation gradient, and strong material anisotropy. The results show that the proposed method can realize the identification of SFC growth morphology, the measurement of crack length, and the measurement of deformation fields near the crack tip region of Q&P steel. The FCG rates, the evolution law of SFC morphology, and crack-tip deformation fields of Q&P steel at different stress ratios were obtained. (1) In the SFC stage of Q&P steel, the increase of the FCG rate is not obvious with the increase of cycle number. The smaller the stress ratio is, the slower the FCG rate is. (2) At the minimum cyclic load, the SFC tip is closed. Only when a certain load is reached does the SFC tip of Q&P steel open. With the load increases, the SFC morphology moves from being slightly opened to fully opened, which is consistent with the crack-closure law. At the same stress ratio, the longer the SFC, the weaker the closing behavior of the SFC. At the same short crack length, the smaller the stress ratio, the stronger the closing behavior of SFC. (3) In a cyclic load, the SFC tip is closed, and the vertical displacement and strain fields are basically not observed. Only when the SFC tip is opened do the vertical displacement and strain fields change significantly. This indicates that the crack-closure effect has a significant influence on the evolution of the vertical displacement and strain fields at the crack tip of Q&P steel. With the load increases, the strain gradually becomes larger. The vertical strain during loading and unloading is basically symmetrical. The evolution law of SFC tip morphology and strain fields in Q&P steel is consistent with the crack-closure law. The method provides experimental and theoretical support for the further study of the SFC growth mechanism and fatigue life prediction of Q&P steel.

## Figures and Tables

**Table 1:** Mechanical properties for Q&P980.

Young’s Modulus, *E* (GPa)	Poisson Ratio, *υ*	Yield Strength, *σ* (MPa)
**196**	0.3	753

**Table 2:** Main hardware parameters.

Hardware	Model	Main Parameters
Camera	BFS-U3-200S6M-C	Resolution: 5472 (H)×3648(V) Sensor area: 1″ Maximum frame rate: 18 fps Minimum exposure time: 2 μs Pixel size 2.4 μm
Microscope lens	Resolv4K	Focal distance: 8.1 mm~9.7 mm Maximum frame rate: 4/3″ Working distance: 10 mm
Camera	acA4112-30 µm	Resolution: 4096(H)×3000(V) Sensor area: 1.1″ Maximum frame rate: 30 fps Minimum exposure time: 2 μs Pixel size: 3.45 μm
Microscope lens	Rodagon 5.6/105 metal	Focal distance: 106.4 mm Maximum imaging circle diameter: 104 mm Working distance: adjusted to <192 mm with a connecting ring

**Table 3:** Fatigue crack’s growth-test parameters.

Stress Ratio	Minimum Load (kN)	Maximum Load (kN)	Frequency (Hz)
0.2	0.45	2.25	8
0.4	1.2	3	8

**Table 4:** The comparison results of crack length.

	1	2	3	4	5	6	7	8	9	10
The algorithms (mm)	0.374	0.558	0.802	1.143	1.457	3.236	5.307	7.412	9.761	14.546
Microscope (mm)	0.381	0.564	0.807	1.148	1.461	3.316	5.367	7.452	9.781	14.576
Relative error (%)	1.837	1.064	0.620	0.436	0.274	2.413	1.12	0.537	0.204	0.206

## Data Availability

Not applicable.
